# Influence of Genotype on Growth Performance, Carcass Traits and Meat Quality: A Comparative Study in Male Alpine and Saanen Kids

**DOI:** 10.3390/ani16060969

**Published:** 2026-03-20

**Authors:** Harun Kutay, Murat Durmuş, İslim Polat Açık, Ugur Serbester

**Affiliations:** 1 Department of Animal Science, Faculty of Agriculture, Cukurova University, 01330 Adana, Türkiye; userbester@cu.edu.tr; 2 Department of Animal Science, Faculty of Agriculture, Tokat Gaziosmanpasa University, 60000 Tokat, Türkiye

**Keywords:** chevon, *Longissimus lumborum*, carcass composition, fatty acid profile, mineral content, amino acid

## Abstract

This study evaluated the growth performance, carcass characteristics, and meat quality of male Alpine and Saanen goat kids raised under standardized fattening conditions to inform breed-specific strategies for meat production. The study included 36 single-born male kids (18 Alpine and 18 Saanen purebreds) of similar age and live weight. The animals were allocated by breed and randomly assigned to three replicates per breed, with six animals in each replicate. After a two-week adaptation period, the kids were fattened for 12 weeks on an 80:20 concentrate–roughage diet. At the end of the fattening period, all animals were slaughtered, and physical, sensory, and chemical analyses of the meat were performed on the *Longissimus lumborum* (LL) muscle. Final live weights did not differ significantly between Alpine and Saanen kids, nor did average daily gains. However, the feed conversion ratio favored the Saanen breed, indicating superior feed efficiency. Alpine kids had significantly higher internal fat content, while Saanen kids had a higher proportion of full intestines. Meat pH, color, and texture were similar between breeds. Fatty acid analysis showed that Alpine meat had higher palmitic and palmitoleic acid content, whereas Saanen meat contained more calcium and sodium. These results suggest that both breeds are suitable for high-quality meat production under controlled feeding conditions, but breed-specific differences in fat composition and mineral content may affect nutritional value and market positioning. These findings are valuable for optimizing selection and marketing strategies in goat meat production systems targeting diverse consumer demands.

## Simple Summary

This study evaluated growth performance, carcass traits, and meat quality of Alpine and Saanen goat kids raised under identical feeding and management conditions. Although no statistically significant differences were detected between breeds, Alpine kids tended to show higher average daily gain and better feed conversion efficiency than Saanen kids, suggesting a potential breed-related advantage in growth performance under the experimental conditions. Carcass characteristics were mainly influenced by slaughter weight, and only minor differences were observed between breeds. Meat quality parameters, including pH, color, tenderness, and chemical composition, were largely similar, indicating comparable meat quality. However, differences were detected in the fatty acid profile, with Alpine kids having higher levels of some saturated and monounsaturated fatty acids. Overall, both breeds produced meat of acceptable quality, while Saanen kids showed advantages in growth efficiency.

## 1. Introduction

Goat meat, also known as chevon, is increasingly appreciated worldwide due to its favorable nutritional profile, its low intramuscular fat content and its special organoleptic properties. It is considered a rich source of high-quality protein, essential fatty acids and important micronutrients such as iron, zinc and B-group vitamins [1]. Moreover, the adaptability of goats to a variety of climatic and nutritional conditions is suitable for meat production in different geographical regions [2]. However, to maximize both meat yield and meat quality, tailored feeding and fattening strategies need to be applied for each breed, as genetic predisposition and environmental conditions significantly influence growth rate, fat deposition patterns and carcass characteristics.

The composition of meat is a decisive factor for the overall quality and has a direct influence on consumer preferences and the profitability of producers. Basic components such as crude protein, lipid content, texture (especially chewiness) and mineral content determine not only the nutritional value but also the sensory properties of meat [3]. A high crude protein content often corresponds to an improved amino acid profile and contributes positively to meeting human nutritional requirements, while a low intramuscular fat content is generally preferred by health-conscious consumers. In addition, the physical texture of meat, especially chewiness, plays a central role in consumer satisfaction and purchasing behavior [4]. Therefore, evaluation of such traits under standardized fattening protocols is essential to align production results with market expectations and achieve commercial success.

Among the dairy-oriented breeds, the Alpine and the Saanen goat are promising candidates for meat production studies, as they have favorable production traits and can adapt to intensive breeding systems [5]. The Alpine breed, originally from the French Alps, is known for its high milk yield, resilience, and ability to perform under different feeding conditions [6]. The Saanen breed, native to Switzerland, is also frequently used in commercial milk production due to its high productivity and docility. Although both breeds are traditionally used for milk production, their suitability for controlled fattening and meat production has attracted increasing interest. However, direct comparative studies focusing on their growth performance, carcass traits and meat quality remain scarce, highlighting the need for further research to support breed-specific optimization strategies [5].

While the fatty acid composition of the meat of these dairy breeds has only been researched to a limited extent, comprehensive analyses of their amino acid profiles are largely lacking. Considering that fatty acid distribution in muscle and adipose tissue serves as a key indicator of meat quality [7], and given the low intramuscular fat content and narrow fatty acid spectrum typical of goat meat [8], this species offers promising potential for the production of lean, high-quality meat. In addition, the distinct fatty acid profile of goat meat makes it an attractive option for health-conscious consumers [9]. Nonetheless, the composition of amino acids remains just as crucial to its nutritional value. Essential amino acids such as lysine, histidine and methionine, which are important for cognitive and physiological development but are not endogenously synthesized by humans, are present in considerable amounts in goat meat [10].

We hypothesized that Alpine and Saanen kids, despite being primarily dairy breeds, show significant differences in fattening performance, carcass traits, and meat quality parameters, especially in amino acid and fatty acid composition, when reared under standardized fattening conditions. The aim of this study is therefore to compare the fattening performance, carcass characteristics and meat quality of male Alpine and Saanen kids reared under similar conditions.

## 2. Material and Methods

### 2.1. Experiment Area

**Figure 1:**
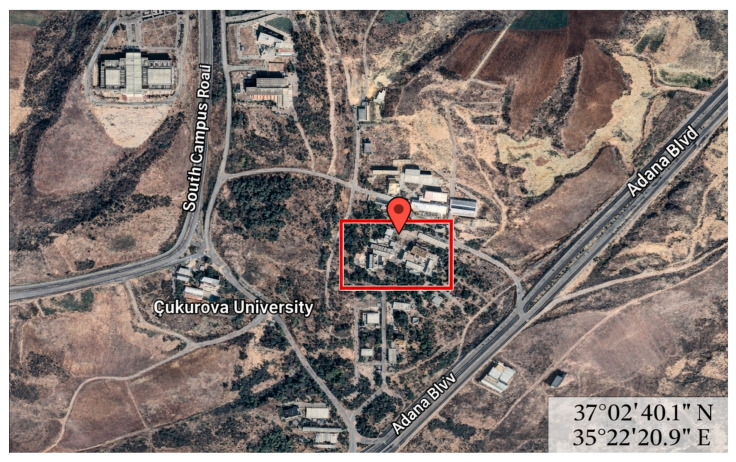
Satellite image and coordinates of the study area [11].

The present study was conducted in the eastern Mediterranean (37°02′ N, 35°22′ E), where subtropical climatic conditions prevail (Figure 1). The animals were housed in six pens, each measuring 9 m^2^ (3.00 × 3.00 m, length × width), with six animals per pen throughout the experimental period. The pens faced each other, and sufficiently large feeders and drinkers were provided for each pen. Urine and feces that accumulated on the concrete floor inside the compartments were cleaned at weekly intervals while the animals were weighed.

### 2.2. Animals, Study Design and Diet

The animal material used in this study, Alpine and Saanen kids, belongs to the Cukurova University, Faculty of Agriculture, Dairy Goat and Ewe Breeding Research and Application Unit, and all necessary official permissions were obtained from the relevant institution prior to the study. This study was approved by the local ethics committee for animal experiments of Çukurova University (approval number: 2024/10). The study included 36 single-born male kids (18 Alpine and 18 Saanen purebreds) of similar age and live weight (14.8 ± 2.73; mean ± standard deviation). The animals were grouped by breed and randomly assigned to three replicates per breed, with six animals in each replicate. Kids were fed primarily on milk and roughage such as alfalfa until weaning, with small amounts of concentrate feed. During the fattening period, a gradual transition was implemented to allow kids to adapt to the ration composition and maintain rumen health, as they would be fed large amounts of concentrate feed. Therefore, a two-week acclimatization phase was carried out before the experiment. In the first week, the animals were fed 60% concentrate and 40% roughage; this ratio changed to 70:30 in the second week and to 80:20 for the remaining 10-week trial period. Throughout the trial, the animals were fed alfalfa hay and concentrate ad libitum. The nutrient composition of these feedstuffs is shown in Table 1.

Nutrient analyses such as dry matter, crude ash, crude protein, ether extract and crude cellulose of the feed used in animal feeding were carried out according to the method in AOAC [12]. In addition, ADF and NDF analyses were performed with the Ankom Fiber Analyzer (ANKOM Technology Corp., Fairport, NY, USA) using the filter bag technique, as reported by Van Soest [13].

### 2.3. Measurements and Analytical Methods

To compare the performance of the breed, feed intake and live weight were recorded. Feed consumption was calculated weekly by subtracting the remaining amount of feed from the total amount of feed administered by weighing it every morning for seven days using a 0.01 g precision digital scale (QW-R, Dikomsan, Ankara, Türkiye). The feed conversion ratio was determined by dividing the dry matter intake by the average daily gain. The live weights of the animals were measured individually every two weeks using a 50 g precision scale (EKO-600, TEM, Ankara, Türkiye). At the end of the trial, all kids were transferred to a commercial slaughterhouse in accordance with European Community laws on animal welfare during transport (Regulation EC 1/2005) and the provisions of the European Community regulation on animal welfare for the slaughter of commercial animals (Regulation EC 1099/2009). In Türkiye, due to religious rules and cultural practices, anesthesia or stunning is not used in animal slaughter, which is performed by a trained and authorized person in accordance with the halal slaughter method. Accordingly, the animals were properly secured before slaughter, and the procedure was performed using a knife with an extremely sharp, smooth blade. The trachea and esophagus, along with both carotid arteries and the jugular vein, were cut in a single, uninterrupted stroke; the spinal cord was not disturbed. No mechanical/electrical stunning or anesthesia was applied during slaughter; the aim was for loss of consciousness to occur as a result of physiological processes due to the sudden interruption of brain perfusion. Throughout the procedure, care was taken to minimize tissue trauma and ensure free blood flow, and the animal was observed until it became completely exsanguine. After complete exsanguination and death, the animals were skinned and their internal organs were removed by an expert person on the team. Then, the weight of the hot carcass as well as the weight of the head, skin, feet, tallow, certain visceral organs (heart, lungs, liver and spleen), the full and empty digestive system and the rumen were recorded. The carcasses were then chilled at +4 °C for 24 h. After chilling, the weight of the cold carcass was measured. By dividing the weight of the cold carcass by the slaughter weight, cold carcass yields (%) were calculated [14]. The testicles and kidneys were then removed and weighed separately, and the diameter of the testicles was measured at the same time. Testicular diameters were determined by measuring the distance between the widest points of each testicle along the horizontal axis with a digital caliper and calculating the average. The left portion of the LL muscle, extending from the last thoracic vertebra (T13) to the last lumbar vertebra (L6), was separated from the carcass to obtain the LL muscle. In these samples, muscle width and depth were measured on the cross-sectional surface at the level of the last rib using a digital caliper (500-716-20, Mitutoyo Corp., Kawasaki, Japan). Muscle width was defined as the maximum horizontal distance and muscle depth as the maximum vertical distance of the muscle section. The fat thickness of the LL was determined by measuring the vertical distance between the dorsal surface of the sample and the outer border of the subcutaneous fat layer with the samedigital caliper.

The physical properties of the meat were evaluated using samples of left LL muscle by pH measurement, cooking loss, color and texture profile analysis (TPA). The pH measurement of meat samples was taken at the 24th hour postmortem. The acidity of the meat was assessed with a portable meat pH meter (HI 99163, Hanna Instruments, Woonsocket, RI, USA; pH: 0–14 range) fitted with an FC 232D electrode [15]. The pH value was calculated by averaging the measurements taken from the proximal, medial, and distal portions of the LL muscle. Cooking loss was determined using a method similar to that described by Fabre et al. [16]. Meat samples were stored at +4 °C for 24 h then allowed to reach room temperature. Raw meat samples were weighed on a scale accurate to 0.001 g then cooked on a double-sided contact electric grill heated to 200 ± 20 °C until the internal temperature reached 74 °C. After cooking, the meat was cooled to room temperature and weighed again on the same scale to determine the cooked weight. Cooking loss was calculated using the following formula.

$$Cooking\;loss\left(\%\right)=\frac{weight\;of\;uncooked\;meat\;sample\;-\;weight\;of\;cooked\;meat\;sample}{weight\;of\;uncooked\;meat\;sample}\;\times \;100$$

A hand-held colorimeter (CR-400, Konica Minolta Inc., Tokyo, Japan) was used to measure the color of the cooked LL muscle meat, and 3 measurements were taken on the outer surface of the uncooked samples. The colorimeter was equipped with an 8 mm aperture and was calibrated with a standard white tile (Y = 93.7, x = 0.3157, y = 0.3323) using illuminant D-65 and 2° observer settings. The color values L*, a*, and b* (L*: brightness; a*: redness; and b*: yellowness) were determined to register the color of the samples as numerical values in the Hunterlab color system (Hunter Associates laboratory Inc., Virginia, USA). Warner–Bratzler shear force values were determined using 3 round core samples (1.27 cm diameter) obtained parallel to the longitudinal orientation of the fibers from uncooked LL muscle samples. In addition, hardness, adhesiveness, cohesiveness, chewiness, resilience and springiness were determined by texture profile analysis (TPA) using 3 round core samples (1.27 cm diameter, 1 cm height) of the cooked meat samples. The properties evaluated by TPA are defined below according to the definition of Paredes et al. [17].

Texture analysis was performed using a texture analyzer (TA-XT Plus, Stable Microsystems, London, UK) with a 50 mm aluminum probe. Samples were compressed to 50% of their height in a double compression cycle at speeds of 1.0 mm/s (pre-test), 2.0 mm/s (test) and 10.0 mm/s (post-test).

The nutrient analyses of the meat included dry matter, crude ash, crude protein and ether extract. Dry matter and crude ash were determined according to the method in AOAC [12], protein according to the Kjeldahl method in AOAC [12] and ether extract according to the method of Bligh and Dyer [18].

Fatty acid methyl esters (FAMEs) were prepared from extracted lipids according to the method of Ichihara et al. [19]. For this purpose, 25 mg of fat was mixed with 4 mL of 2 M KOH and 2 mL of *n*-heptane, vortexed for 2 min and centrifuged at 4000 rpm for 10 min. After centrifugation, the upper heptane layer was transferred to vials for analysis by gas chromatography (GC). Analysis of fatty acids in the heptane layer was performed using an Agilent GC-MS/MS (7890B GC–7010B MS, Agilent Technologies Inc., Santa Clara, CA, USA) with an auto sampler (Gerstel GmbH, Mülheim an der Ruhr, Germany) equipped with a flame ionization detector and an Agilent capilllary column (60 m × 0.25 mm × 0.25 µm; DB-WAX capillary column, Agilent Technologies Inc., Santa Clara, CA, USA). The oven temperature was held at 50 °C for 1 min, then increased to 200 °C at a rate of 25 °C/min and held for 10 min, then increased to 230 °C at a rate of 3 °C/min and held at this temperature for 26 min. The injector and detector temperatures were set at 250 °C and 300 °C, respectively. The sample size was 1 µL and the flow rate of the carrier gas was 1mL/min helium. The split used was 1:40, and the fatty acids were identified by comparing the retention times of FAME with the standard 37-component FAME mixture.

The mineral contents (Na, Mg, K, Ca, Fe, Cu, and Zn) of the meat were measured using inductively coupled plasma mass spectrometry (ICP-MS Series 2, Thermo Fisher Scientific, Bremen, Germany) with collision cell technology according to Betancor et al. [20]. Prior to analysis, 25 mg of meat was digested in Teflon tubes with 5 mL of 69% nitric acid (Aristar^®^ analytical grade, VWR Chemicals, Lutterworth, UK) using a microwave digestion system (MARS Xpress, CEM Corporation, Matthews, NC, USA). The ICP-MS operated in kinetic energy discrimination (KED) mode with helium as the collision gas and argon as the plasma gas; scandium and gallium were used as internal standards.

For the determination of the free amino acid content in the meat, extraction was performed with modifications to the method described by Koutsidis et al. [21]. After slaughtering the kids for the determination of the amino acid content in the meat, a 0.1 g sample from the LL muscle of each animal was weighed; 0.9 mL potassium chloride was added and homogenized. Then, the samples were centrifuged at 5000 rpm at +4 °C to obtain the supernatant serum. An amount of 70 µL of the supernatant obtained was transferred to Eppendorf tubes, and 70 µL of the IS MIX mixture was added and vortexed. Then, this mixture was incubated at +4 °C for 15 min and then centrifuged at 10,000 rpm for 5 min. After centrifugation, 100 µL of the mixture was transferred to the supernatant plate and the nitrogen was evaporated at 40 °C. After evaporation of the nitrogen, 50 µL of derivatization reagent was added to the mixture and then incubated in the oven at 60 °C for 15 min. The samples removed from the oven were evaporated under nitrogen at 40 °C, and then 100 µL of solvent was added. Of these solvents, 50% was mobile A (1.58 g of ammonium formate and 2.5 mL of formic acid were added to 250 mL of distilled water), and the other 50% was mobile B (500 µL of formic acid was added to 500 mL of acetonitrile). Finally, the resulting mixture was shaken in the shaker for 5 min and then transferred to the filtered Eppendorf tubes and centrifuged for one minute. Then, 3 µL of the sample added to the insert tube was transferred to the Thermo Fisher Scientific Liquid Chromatography Mass System (LC-MS/MS, Thermo Fisher Scientific, Waltham, MA, USA) instrument for analysis. In the instrument used for the analysis, the capillary temperature (250 °C), evaporator temperature (300 °C), aux gas pressure (15), sheet gas pressure (35), ion sweep gas pressure (0), collision gas pressure (1.4), Spery voltage (5000 V) and column oven temperature (25 °C) were set. In addition, calibration and control studies were performed with the instrument before the samples were analyzed, and all analyses performed were examined with the control.

### 2.4. Statistical Analysis

Data collection and statistical analysis were performed using Microsoft Excel (Microsoft Office Professional Plus 2016, Microsoft, Redmond, WA, USA) and SAS (version 9.4, SAS Institute Inc., Cary, NC, USA), respectively. All analyses were performed on a group basis and are considered from this perspective in the text. Repeated measures were analyzed using a mixed model (PROC MIXED) to assess multiple measured variables such as body weight and feed intake. Initial live weight at baseline was included in the model as a covariate, with repeated weekly measurements and within-breed groups (three groups per breed) treated as random effects. An order 1 autoregressive covariance structure was used [22,23]. For variables that were measured once at the end of the trial (e.g., carcass weight, nutrient analyzes, and minerals), a mixed model was used in which final live weight was included as a covariate and within-breed groups were treated as a random effect. Least squares means and pooled standard errors were reported in the tables. Differences between breeds were evaluated based on the fixed effect of breed in the mixed model. Statistical significance was set at *p* < 0.05, and trends were identified when 0.05 < *p* ≤ 0.10.

## 3. Results

### 3.1. Performance and Carcass Traits

The performance values of the Alpine and Saanen male kids in the experiment are shown in Table 2. Although the final live weight of the Alpine kids was slightly higher than that of the Saanen kids, the difference was minimal and the difference between the means was insignificant (*p* > 0.05). At the end of the present study, it was observed that weaned male Alpine and Saanen kids had similar fattening performance.

The carcass and organ measurements of the kids slaughtered at the end of the 12-week fattening period for Alpine and Saanen male kids are shown in Table 3. When looking at the table, significant (*p* < 0.05) differences were found between the breeds in terms of the proportional weights of full intestine and tallow. The differences between breeds in terms of cold carcass weight (*p* = 0.072), full digestive system weight (*p* = 0.058) and empty rumen weight (*p* = 0.060) were close to statistical significance

### 3.2. Meat Analysis

The quality characteristics of the raw and cooked meat of male Alpine and Saanen kids after slaughter are shown in Table 4. Breed had no significant effect on pH, cooking loss, color or shear force of the raw meat (*p* > 0.05). The effect of breed on the color and texture profile analysis of the cooked meat also proved to be insignificant (*p* > 0.05). In general, it can be said that the breed has no effect on the visual and textural properties of the meat. Neither the male kids of the Alpine breed nor those of the Saanen breed proved to be better than the others in terms of consumer acceptance.

The results of the analysis of the nutrient and fatty acid composition of the meat of the slaughtered Alpine and Saanen kids at the end of the study are presented in Table 5. This table shows that the breeds had no significant (*p* > 0.05) influence on the nutrient composition of the meat. It was found that both breeds were similar in terms of nutrient composition. The amounts of palmitic acid (C16:0) and palmitoleic acid (C16:1) were significantly (*p* < 0.05) higher in the Alpine breed kids. When fatty acids were grouped according to degree of saturation, no significant differences were observed between breeds for total saturated fatty acid (ΣSFA), monounsaturated fatty acid (ΣMUFA), or polyunsaturated fatty acid (ΣPUFA). The ΣSFA content averaged 37.60% in Alpine and 40.96% in Saanen kids (*p* = 0.233), while ΣMUFA accounted for 49.23% and 48.04% of total fatty acids in Alpine and Saanen kids, respectively (*p* = 0.692). Total polyunsaturated fatty acid (ΣPUFA) was similar between breeds (4.69% vs. 4.96%; *p* = 0.765).

The mineral content of the meat samples examined in the study is shown in Table 6. The Ca and Na contents were significantly (*p* < 0.05) higher in the Saanen breed kids than in the Alpine breed. However, no significant differences were found between the breeds with regard to the amounts of other minerals in the meat.

The amino acid composition of the meat of the male kids from Alpine and Saanen kids slaughtered at the end of the study is shown in Table 7. The table shows that the levels of the amino acids 3-methyl-histidine and asparagine were significantly higher in the meat of the Alpine kids, while the cystine content was significantly higher in the meat of the Saanen kids. However, the differences between the breeds with regard to the amounts of other amino acids in the meat of kids were found to be insignificant.

## 4. Discussion

The higher live weight of the Saanen kids compared to the Alpine kids can be attributed to the influence of breed. Previous researchers have also reported that breed has a significant effect on live weight gain [24,25]. The superior growth rate and feed conversion ratio of the Saanen breed may have contributed to this effect. In addition, the higher initial live weight of the Saanen kids (about 0.26 kg more) could also help to explain the observed difference. The live weight values determined in the present study are consistent with the findings reported by Gökdal et al. [26] and Atay [27].

The Alpine kids consumed more feed than the Saanen kids, but only very small differences in live weight gain were observed between the two breeds. Despite the higher feed intake of the Alpine kids, the live weight gains of the Alpine kids are similar compared to those of the Saanen kids, which can be explained by the lower feed conversion rate of the Alpine kids. Previous studies have also shown that Saanen kids have a better feed conversion ratio than Alpine kids [27,28,29]. Therefore, the data obtained in the present study are consistent with the literature and show that the fattening performance of Saanen kids is superior to that of Alpine kids.

Since the slaughter weights of the Alpine kids at the end of the 12th week were numerically higher than those of the Saanen kids, it is to be expected that their hot carcass weights would also be higher. Indeed, previous researchers have found that carcass weight is related to slaughter weight, which is consistent with the existing literature [30,31]. In cold carcasses, it was found that Alpines have a numerically higher weight than Saanens. This effect is due to the already higher weight of the hot carcasses in the Alpine group. In addition, the carcasses must be stored at +4 °C for 24 h after slaughter in order to reach rigor mortis. During this period, there is a loss of moisture through evaporation, resulting in a weight loss of the cold carcass of about 2% [32]. The fact that the difference between the weights of the hot carcasses was not statistically significant, while the weights of the cold carcasses showed an almost significant difference, indicates that the Saanen carcasses suffered a greater moisture loss during the chilling process.

Although the proportionate weight of the full small intestine was higher in the Saanen kids than in the Alpine kids, the proportionate empty weight of the same organ was similar in both breeds. Despite the higher feed intake in the Alpine kids, the higher full weight of the small intestine in the Saanen kids could be related to the length of the digestive tract or the speed of passage through the digestive system. Feed and water were removed from the animals one day before slaughter. Nevertheless, the observed differences in the weight ratio of the small intestines between breeds support this hypothesis. Similarly, Campos et al. [33] reported that the content of the digestive system varied between breeds. When the proportional weights of internal fat were examined, the values for Alpine kids were significantly higher than those of Saanen. It was found that dairy goat breeds tend to store more fat in the internal organs than in the carcass fat [34]. This could indicate that the Alpine kids used in the study have stronger milk characteristics compared to the Saanen in this trial. No significant differences were found between the breeds with regard to the weight ratio of the other internal organs. Similar results have also been reported by other researchers [26,35].

The pH value of the meat is related to the stress to which the animals were exposed before and during slaughter, as well as to the glycogen reserves in the muscles, regardless of many other factors. Under stress, the increased release of adrenaline leads to a rapid depletion of glycogen in the muscles, resulting in an accumulation of lactic acid. The accumulation of lactic acid in the muscles leads to a decrease in the pH of the meat [36]. While the pH of the muscle tissue of a living animal is between 7.0 and 7.2, the pH of meat after lactic acid formation should normally be between 5.6 and 5.9 [37]. In the present study, ultimate pH values ranged from 5.83 to 5.89 and were measured at 24 h postmortem. These values fall within the normal pH range reported for goat meat and are above the threshold commonly associated with PSE conditions (pH < 5.6) and below those indicative of DFD meat (pH > 6.4). pH values below 5.6 are generally associated with PSE-like characteristics, whereas values exceeding 6.4 are indicative of DFD conditions in red meat [38]. The similarities in the determined pH values are in line with the results of Ivanovic et al. [8]. The absence of significant pH differences among breeds may be attributed to the standardized pre- and post-slaughter handling procedures applied to all animals, including similar fasting duration, transport conditions, lairage time, and chilling regimes, which are known to strongly influence postmortem muscle metabolism [39]. If the muscle pH falls below these values after slaughter, this can lead to pale, soft, exudative meat, while an increase above these values leads to dark, firm, dry meat. This in turn affects the color and water-holding capacity of the meat [38]. In the present study, since the pH values were similar, no differences were found in cooking loss and color values (L*, a*, and b*). Similarly, Ivanovic et al. [8] found that the color measurements of Alpine and Saanen meats were quite similar.

The structural properties of meat and its perceived texture in the mouth are of great importance for consumer acceptance. These properties are influenced by factors such as breed, age, sex, feeding and management conditions. For example, Monsón et al. [40] reported that beef from cattle has a lower shear force than that from dairy cows. Similarly, Sañudo et al. [41] found that breed has an influence on meat quality. In the current study, male kids of similar age and live weight were fed the same feed under the same conditions. As both breeds are dairy breeds, the lack of differences in texture profile between the groups could be attributed to this factor. Atay [27] also found that shear force in Alpine and Saanen crossbreeds was not influenced by breed and was similar. Similarly, Dhanda et al. [42] indicated that shear force was not significantly influenced by breed.

Meat consists of nutrients such as water, protein, fat and carbohydrates, which animals accumulate in their bodies to increase their body mass. The nutrient composition of meat varies depending on factors such as genotype, sex, age, management and feeding practices [43]. The proteins in muscle mass are the result of protein synthesis from actin and myosin in the ribosomes of myocytes [44]. The amino acids required for this protein synthesis come from exogenous (dietary) or endogenous (microbial) protein sources [45]. In this study, male kids of different genotypes (Alpine and Saanen) but similar ages were fed the same type of ration under identical environmental conditions. Therefore, it can be said with certainty that the intake of exogenous amino acids was similar between the breeds. Furthermore, the similar protein levels in the meat indicate that microbial protein synthesis (an endogenous amino acid source) did not differ between the breeds. Fat in muscle is in the form of triglycerides, which are formed by combining fatty acids taken into the body with glycerol, which is produced from glucose in the liver and myocytes [46]. In addition, excess energy intake is first converted to glycogen and then to acetyl-CoA for lipogenesis [47]. Similarly, acetate formed in the rumen is also converted to acetyl-CoA and used for lipogenesis [48]. The lack of significant differences in the fat content of the meat in this study can be attributed to similar fatty acid and energy intake via the ration and the lack of differences in fat synthesis between breeds. As also noted by Atay [27], no significant differences were found between breeds in terms of the nutrient composition of the meat in this study. However, another study reported that the nutrient composition of the meat is significantly influenced by the breed of kids [8]. The researchers explained that it is quite difficult to explain such differences due to the limited number of studies that have investigated meat quality in different breeds of dairy goats bred for slaughter.

The fatty acid profile of meat is partly influenced by diet, but mainly by genetic factors [49]. The main sources of fat for ruminants are dietary fat and microbial fat. Therefore, the differences in the fatty acid profile of the meat in this study, which were due to the use of a uniform diet, were influenced by microbial fermentation in the rumen or genetic factors. Similar to the present results, several researchers have found that the fatty acid profile of meat changes depending on the breed [7,31,50]. Based on the results, it can be said that kids of the Alpine breed have a higher synthesis of palmitic and palmitoleic acids compared to kids of the Saanen breed.

However, it is known that myristic acid and palmitic acid increase blood cholesterol levels in humans [51]. From this point of view, it could be argued that Saanen meat is potentially healthier. Palmitoleic acid, one of the most abundant fatty acids in animal fats, is formed by the desaturation of palmitic acid [52]. This conversion occurs in the liver and adipose tissue by the enzyme stearoyl-CoA desaturase [49,53]. In addition, palmitoleic acid is also produced by the breakdown of carbohydrates by microorganisms such as *Eubacterium ruminantium* in the rumen [54,55]. Although not definitive, it can be hypothesized that the rumen microbiota of Alpine goats differs from that of Saanen goats or that palmitoleic acid synthesis is more efficient in Alpine goats. However, more detailed microbiological studies are needed to confirm this hypothesis. Apart from palmitoleic acid, the content of other unsaturated fatty acids in meat, which are of great importance for human health, was not significantly influenced by breed. Since the experimental diet was predominantly cereal-based, it can be considered rich in omega-6 fatty acids and relatively poor in omega-3 fatty acids. Although the ether extract content of the meat samples was at a satisfactory level, the low omega-3 content of the diet may not have been reflected in the muscle tissue. Consequently, omega-3 fatty acids may have remained below the detection range of the analytical instrument and therefore could not be detected. In fact, some researchers have reported trace levels of omega-3 fatty acids in kid meat (<0.4 g/100 g) [56,57]. Furthermore, despite the relatively higher omega-6 content of the diet, its concentration in the muscle may have decreased due to ruminal biohydrogenation, resulting in levels below the detectable range of the device. Considering the total fatty acids, there appears to be a loss of 7.86% in Alpine and 6.61% in Saanen kids. This loss is thought to occur because small amounts of fatty acids remain below the device’s detection range.

Calcium, an essential building block of mammalian organisms, is the most abundant mineral in the body. It plays a key role in the formation of the skeletal system and teeth, muscle contraction and blood clotting. Sodium, on the other hand, is responsible for maintaining acid–base balance, facilitating nerve and muscle function, and aiding glucose uptake. Mineral content in goat meat has been reported to vary significantly depending on the breed [58,59,60]. While calcium is concentrated in bones and teeth among body tissues, its content in meat is relatively low. However, calcium is indirectly involved in the tenderness of meat due to its role in postmortem calpain and calpastatin synthesis [61]. It has also been suggested that calcium content in meat may be related to a single-nucleotide polymorphism in the CAPN1 gene [61]. Unfortunately, because gene expression was not examined in this study, it is difficult to express this difference between breeds. Detailed studies examining the interaction of genes responsible for calcium and its storage in meat would add depth to future studies. Studies on the sodium content of meat generally focus on overall mineral composition, and research looking directly at the effects of breed on sodium content is limited. However, sodium content has been reported to vary depending on factors such as breed, feeding method, sex and the specific part of the meat from which the sample was taken [62,63].

It is said that there are more than 700 amino acids in nature, but 20 of them are important as building blocks of proteins in cells [64]. Goat meat is one of the richest sources of essential amino acids such as threonine, lysine and tryptophan, which are of great importance for human nutrition [65]. The amino acid composition of meat can vary depending on breed, age, sex, diet, physiological state and hormones, as well as post-slaughter processes. Previous researchers have also found that the amino acid composition of goat meat varies by breed [8,10,66].

The amino acids in meat are synthesized by the binding of ammonia (amination) or the addition of an amino group (transamination) to the carbon skeleton formed by glycolysis, the Krebs cycle or the pentose–phosphate pathway in myocytes [67]. The amino acids required for muscle synthesis are absorbed via the intestine and transported to the myocytes. These sources are provided by amino acids that either bypass the rumen or are formed in the rumen during microbial fermentation. The amino acids that reach the myocytes are assembled into muscle proteins in the ribosomes under the influence of hormones such as growth hormone, testosterone and insulin under the guidance of mRNA. In the current study, male goat kids of similar age and weight, belonging to two different breeds, were fed the same diet. Therefore, the differences in the amount of some amino acids in the meat can be attributed to the influence of breed. However, to explain this situation more precisely, it is necessary to measure the mRNA activity, the amount and variety of amino acids reaching the intestine, and the hormone activities involved in muscle synthesis. On the other hand, when total amino acids were considered, losses of 1.905% in Alpine and 6.087% in Saanen kids were observed. These losses are presumed to result from trace amounts of certain amino acids falling below the detection ranges of the analytical instrument.

## 5. Conclusions

This study evaluated the influence of breed on growth performance, carcass traits, and meat quality characteristics of Alpine and Saanen male kids reared under identical housing and feeding conditions. Under these controlled conditions, overall growth performance and most general meat quality parameters, including pH, color, cooking loss, and texture, were largely comparable between breeds. However, the results indicate that breed effects were not negligible but rather trait-specific. Notable breed-related differences were observed in feed efficiency, carcass composition, and biochemical characteristics of the meat. Alpine kids exhibited a numerically better feed conversion ratio and higher concentrations of calcium and sodium in muscle tissue, traits that may be advantageous in intensive meat production systems prioritizing feed efficiency and mineral content. In addition, Alpine kids showed higher internal fat deposition and elevated levels of certain fatty acids (palmitic and palmitoleic acids), as well as higher concentrations of specific amino acids such as 3-methylhistidine and asparagine, which may influence the nutritional value and metabolic characteristics of the meat.

The observed differences in fatty acid and amino acid profiles may be related to genetic factors and/or breed-specific differences in rumen microbial activity; however, these explanations remain hypothetical and should be interpreted with caution, as direct measurements of rumen microbiota composition or gene expression were not included in the present study. Future studies integrating molecular, microbiological, and metabolic approaches would be required to clarify the underlying mechanisms. Several limitations of this study should also be acknowledged, including the relatively limited sample size, the narrow age and weight range of the animals, and the single fattening duration evaluated. These factors may have constrained the magnitude of detectable breed differences and should be considered when extrapolating the results to other production systems. In conclusion, while Alpine and Saanen kids produced meat of generally comparable quality under standardized conditions, breed-related differences were evident in specific performance, carcass, and compositional traits. These findings suggest that breed selection in meat-oriented goat production systems should be based on targeted production goals rather than overall meat quality alone.

## Figures and Tables

**Table 1:** Ingredients and nutrient compositions of fattening diet.

Ingredients	% of DM
Alfalfa hay	19.88
Corn grain	16.15
Wheat bran	13.66
Barley grain	8.70
Corn bran	16.15
Canola meal	6.21
Soybean meal	4.97
Corn dist ethanol	3.73
Molasses	3.73
Wheat midds	3.73
Limestone	2.48
Salt	0.50
Premix ^1^	0.12
Nutrient composition (% of DM)	
Dry matter	88.9
Crude protein	16.2
Ether extract	3.3
NDF ^2^	35.7
ADF ^2^	17.7
Crude ash	8.91
Ca	1.20
Cu (ppm)	10.27
Fe (ppm)	153.75
K	1.59
Mg	0.42
Na	0.30
Zn (ppm)	64.57
P	0.74
Fat profile mg/100 g feed	
Myristic acid (C14:0)	61.3
Palmitic acid (C16:0)	371.2
Palmitoleic acid (C16:1)	55.3
Heptadecanoic acid (C17:0)	4.6
Stearic acid (C18:0)	165.0
Oleic acid (cis-trans) (C18:1)	501.2
Linoleic acid (cis-trans) C18:2*n*-6	445.7
Linolenic acid (C18:3*n*-3)	32.8

^1^ The premix provided the following per kg of the fattening diet: vitamin A 1367 IU, vitamin D 194 IU, vitamin E 15 IU, Fe 74 mg, Zn 46.3 mg, Mn 36.5 mg, Cu 17.0 mg, I 1.5 mg, Co 0.3 mg, and Se 0.3 mg. ^2^ ADF: Acid Detergent Fiber, NDF: Neutral Detergent Fiber, DM: dry matter.

**Table 2:** Fattening performance of kids fed the experimental diet.

Performance Values	Breed		SEM	95% CI	*p*-Value
	Alpine	Saanen			
Initial live weights (kg)	14.68	12.97	0.85	12.30–15.23	0.242
Final live weights (kg)	23.30	20.91	0.79	20.56–23.65	0.079
Dry matter intakes (kg/day)	0.88	0.81	0.04	0.76–0.91	0.425
Average daily gain (g/day)	122.86	113.57	16.92	91.08–149.29	0.410
Feed conversion ratio	7.53	7.41	1.15	5.62–9.49	0.425

SEM: Standard Error of Means, CI: Confidence Interval.

**Table 3:** Slaughter weight and carcass characteristics of kids.

Variable	Breed		SEM	95% CI	*p*-Value
	Alpine	Saanen			
Slaughter weights (kg)	23.30	20.91	0.79	20.56–23.65	0.079
Hot carcass (kg)	10.50	9.30	0.34	9.23–10.57	0.160
Cold carcass (kg)	9.84	8.47	0.31	8.55–9.76	0.072
Carcass yield (%)	46.40	41.70	1.63	40.86–47.24	0.165
Blood (%)	6.55	6.36	0.85	4.79–8.12	0.896
Skin (%)	6.84	6.01	0.30	5.84–7.01	0.189
Head (%)	6.97	6.38	0.30	6.09–7.26	0.300
Legs (%)	2.75	2.62	0.16	2.37–3.00	0.657
Lungs with trachea (%)	5.46	5.45	0.36	4.75–6.16	0.990
Heart (%)	0.42	0.40	0.02	0.37–0.45	0.599
Lung (%)	1.95	1.88	0.09	1.74–2.09	0.685
Liver (%)	2.53	2.77	0.30	2.06–3.24	0.658
Spleen (%)	0.17	0.17	0.02	0.13–0.21	0.835
Full digestive system (%)	22.27	29.72	1.56	22.94–29.05	0.058
Empty digestive system (%)	7.33	7.99	0.34	6.99–8.33	0.309
Full rumen (%)	8.83	12.76	0.99	8.85–12.74	0.089
Empty rumen (%)	1.24	1.90	0.14	1.30–1.84	0.060
Full intestine (%)	9.02	12.51	0.54	9.71–11.82	0.027 *
Empty intestine (%)	4.65	4.66	0.23	4.20–5.11	0.988
Tallow fat (%)	1.13	0.41	0.08	0.61–0.93	0.013 *
Kidney (%)	0.39	0.43	0.04	0.33–0.49	0.563
Kidney fat(%)	0.61	0.36	0.10	0.29–0.68	0.205
Testis (%)	0.61	0.58	0.04	0.52–0.67	0.773
Testicular diameter (mm)	42.6	43.30	1.10	40.79–45.11	0.708
LL depth (mm)	23.20	22.00	2.24	18.21–26.99	0.763
LL width (mm)	43.70	45.20	1.63	41.26–47.64	0.606
LL fat thickness (mm)	0.43	0.46	0.12	0.21–0.68	0.873

SEM: Standard Error of Means, CI: Confidence Interval, *: The difference between the group means is significant for the measured value (*p* < 0.05).

**Table 4:** Quality of raw and cooked meats of kids.

Variable	Breed		SEM	95% CI	*p*-Value
	Alpine	Saanen			
pH_24_	5.89	5.83	0.04	5.78–5.94	0.415
Cooking loss (g)	17.70	11.30	1.92	10.74–18.26	0.128
Color					
Raw meat					
L*	50.60	52.70	1.05	49.59–53.71	0.300
a*	13.40	12.30	0.88	11.13–14.57	0.462
b*	6.42	6.22	0.75	4.85–7.79	0.877
Cooked meat					
L*	61.80	64.10	0.83	61.32–64.58	0.168
a*	7.170	7.11	0.57	6.02–8.26	0.953
b*	11.70	12.20	0.33	11.30–12.60	0.372
Texture Profile Analysis					
Raw meat					
Shear force (kg/cm^2^)	7334.10	6561.60	520.20	5928.26–7967.44	0.420
Cooked meat					
Hardness	3576.10	3566.50	823.2	1957.83–5184.77	0.995
Chewiness	926.60	814.10	234.2	411.32–1329.38	0.783
Cohesiveness	0.571	0.510	0.03	0.48–0.60	0.248
Gumminess	1970.00	1823.90	405.8	1101.58–2692.32	0.836
Resilience	0.195	0.181	0.01	0.17–0.21	0.146
Springiness	0.452	0.414	0.03	0.37–0.49	0.483

SEM: Standard Error of Means, CI: Confidence Interval.

**Table 5:** Meat nutrient content and fatty acid profiles of kids.

Variable (%)	Breed		SEM	95% CI	*p*-Value
	Alpine	Saanen			
Dry matter	28.90	27.60	0.78	26.72–29.78	0.378
Crude protein	24.30	25.30	0.67	23.49–26.11	0.423
Ether extract	4.97	4.30	0.76	3.15–6.12	0.598
Ash	1.96	1.23	0.50	0.62–2.58	0.369
Fatty acid profile (% of fatty acids)					
Myristic acid (C14:0)	2.52	1.48	0.24	1.53–2.47	0.074
Pentadecanoic acid (C15:0)	0.60	0.42	0.09	0.33–0.69	0.300
Palmitic acid (C16:0)	23.50	19.70	0.71	20.21–22.99	0.044 *
Palmitoleic acid (C16:1)	3.08	1.96	0.22	2.09–2.95	0.048 *
Heptadecanoic acid (C17:0)	1.58	1.34	0.36	0.75–2.17	0.701
cis-10 Heptadecanoic acid (C17:1)	1.94	1.56	0.37	1.02–2.48	0.569
Stearic acid (C18:0)	10.60	16.90	2.00	9.83–17.67	0.145
Oleic acid (cis-trans) (C18:1)	43.40	45.30	2.46	39.53–49.17	0.667
Linoleic acid (cis-trans) (C18:2)	3.97	3.52	0.50	2.77–4.73	0.613
Arachidonic acid (C20:4*n*-6)	0.95	1.21	0.32	0.45–1.71	0.633
SFA	37.60	40.96	1.69	35.97–42.60	0.233
MUFA	49.23	48.04	1.97	44.77–52.49	0.692
PUFA	4.69	4.96	0.62	3.62–6.03	0.765

SEM: Standard Error of Means, CI: Confidence Interval, *: The difference between the group means is significant for the measured value (*p* < 0.05).

**Table 6:** Meat mineral matter contents of kids (ppm).

Variable	Breed		SEM	95% CI	*p*-Value
	Alpine	Saanen			
Ca	154.00	280.00	50.7	200.48–233.52	0.003 *
Cu	1.41	1.45	0.16	1.12–1.74	0.914
Fe	21.30	21.90	1.33	18.99–24.21	0.806
K	5013.60	5448.80	278	4686.32–5776.08	0.399
Mg	331.00	359.60	24.9	296.50–394.10	0.565
Na	955.80	1218.40	36.3	1015.95–1158.25	0.020 *
Zn	55.90	54.70	9.31	37.05–73.55	0.943

SEM: Standard Error of Means, CI: Confidence Interval, *: The difference between the group means is significant for the measured value (*p* < 0.05).

**Table 7:** Meat amino acid composition of kids (g/100 g).

Variable	Breed		SEM	95% CI	*p*-Value
	Alpine	Saanen			
1-Methyl_histidine	0.098	0.09	0.009	0.08- 0.11	0.571
3-Methyl_histidine	0.218	0.137	0.019	0.14–0.21	0.025 *
Alanine	24.9	22.2	1.43	20.75–26.35	0.229
α-Aminobutyric acid	1.21	1.40	0.354	0.61–2.00	0.718
Arginine	1.83	2.00	0.063	1.79–2.04	0.096
Asparagine	0.773	0.604	0.047	0.60–0.78	0.043 *
Aspartic acid	0.289	0.295	0.042	0.21–0.37	0.928
Citrulline	0.151	0.167	0.023	0.11–0.20	0.643
Cystine	0.025	0.028	0.0005	0.03–0.03	0.010 *
Glutamic acid	3.73	3.47	0.537	2.55–4.65	0.742
Glutamine	17.6	13.2	1.82	11.83–18.97	0.142
Glycine	25.8	28.3	4.00	19.21–34.89	0.676
Histidine	0.361	0.308	0.057	0.22–0.45	0.535
Hydroxy-L-Proline	0.359	0.313	0.03	0.28–0.39	0.317
Isoleucine	0.762	0.866	0.064	0.69–0.94	0.293
Leucine	1.07	1.14	0.085	0.94–1.27	0.568
Lysine	1.06	0.939	0.078	0.85–1.15	0.326
Methionine	0.227	0.311	0.041	0.19–0.35	0.193
Ornithine	0.861	0.821	0.139	0.57–1.11	0.848
Phenylalanine	0.403	0.484	0.04	0.37–0.52	0.203
Proline	1.26	1.21	0.083	1.07–1.40	0.645
Serine	1.67	2.23	0.346	1.27–2.63	0.296
Taurin	10.2	10.6	1.15	8.15–12.65	0.816
Threonine	1.42	0.773	0.246	0.61–1.58	0.114
Tryptophan	0.144	0.192	0.031	0.11–0.23	0.308
Tyrosine	0.544	0.585	0.029	0.51–0.62	0.365
Valine	1.13	1.25	0.076	1.04–1.34	0.303

SEM: Standard Error of Means, CI: Confidence Interval, *: The difference between the group means is significant for the measured value (*p* < 0.05).

## Data Availability

Data can be made available on request due to restrictions (e.g., privacy, legal or ethical reasons).
